# Growth until Peak Height Velocity Occurs Rapidly in Early Maturing Adolescent Boys

**DOI:** 10.3390/children9101570

**Published:** 2022-10-18

**Authors:** Toshiharu Tsutsui, Satoshi Iizuka, Wataru Sakamaki, Toshihiro Maemichi, Suguru Torii

**Affiliations:** 1 Faculty of Sport Sciences, Waseda University, 2-579-15 Mikajima, Tokorozawa, Saitama 359-1192, Japan; 2 Japan Institute of Sports Science, 3-15-1 Nishigaoka, Kita-ku, Tokyo 115-0056, Japan; 3 Graduate School of Sport Sciences, 2-579-15 Mikajima, Tokorozawa, Saitama 359-1192, Japan

**Keywords:** biological maturity status, growth curve, growth tempo, youth athlete

## Abstract

The timing and tempo of growth rate varies inter-individually during adolescence and can have an impact on athletic performance. This study aimed to determine the difference in growth rate for each maturity status. We combined data collected both retrospectively and prospectively from 78 adolescent boys aged 12 years old; growth charts were collected from their elementary school records, and the height of each participant was subsequently measured every six months over a period of two years. Take Off Age (TOA), Peak Height Velocity Age (PHVA), and Final Height Age (FHA) were estimated using the AUXAL 3.1 program. Growth Tempo 1 and 2 were calculated by dividing the height increase by the time difference between TOA and PHVA, and FHA, respectively. Our results showed three group differences based on the maturation status of PHVA: Growth Tempo 1 and 2 were both higher in the early than during middle and late maturation. Additionally, entering the height at each event as a covariate, the group differences for Growth Tempo 1 did not change; however, for Growth Tempo 2, group differences were eliminated. Therefore, we conclude that during early maturation, growth from TOA to PHVA occurs rapidly and in a shorter period.

## 1. Introduction

Adolescent growth spurt refers to the period of height increase that occurs concomitant with the development of secondary sexual characteristics. The time when the annual height increase is maximized is called the peak height velocity age (PHVA); it usually occurs around 13–15 years of age for boys and 11–12 years of age for girls [1]. Various changes occur in the musculoskeletal apparatus around PHVA, such as an increase in muscle mass [2,3] and tightness [4], and a temporary decrease in bone mineral density [5].

This growth process varies between individuals [6], and it can be evaluated with respect to timing and tempo. The methods used to evaluate the “timing” of the growth spurt in young athletes include peak height velocity (PHV), menarche in girls [1], maturity offset predicted by anthropometric measurement [7] or “status” (which includes skeletal age) [8,9] and percentage of predicted adult height [10]. However, the “tempo” of growth is scarcely reported in the literature due to the difficulty of collecting longitudinal data.

The process of growth is known to increase the risk of injuries [11] and impede athletic performance improvement [12]. The timing of maturation varies greatly among individuals, even if they are of the same age. In contact sports, this results in a mismatch, where late-maturing individuals are relatively inferior physically to those who mature earlier. The effects of such a mismatch can be of concern, particularly in games involving physical contact; hence, many reports have focused on the timing of maturation in soccer [13] and American football [10]. To address this mismatch, some studies [6,14] have reported the concept of bio-banding, which involves grouping young athletes by their individual differences with respect to size, strength, and power.

While both Wik et al. [15] and Steidl-Müller et al. [16] have stated that rapid growth is associated with the occurrence of injuries, it is unclear at what ages the growth processes in these studies were evaluated, as they used annual increase in height as the growth measure. Considering that long-term longitudinal investigation is required to calculate growth tempo, maturity status should be considered when evaluating the occurrence of injuries or improvement of athletic performance.

To determine the best time for interventions to prevent growth-related injuries or improve athletic performance in adolescence, a better understanding of the timing of the growth tempo is needed. Therefore, this study aimed to investigate the differences in growth tempo with respect to maturity status—early, middle, and late.

## 2. Materials and Methods

### 2.1. Subjects

A cohort of 100 Japanese male adolescent soccer players, aged 12 years, belonging to a town recreation soccer team in Tokyo were recruited for this study. They played regular soccer practices or games after school and on weekends. After, we applied two exclusion criteria: (i) no injuries or diseases and delayed puberty or hypogonadism affecting the physical growth with limitations of daily life—such as fracture or epiphysiolysis, and (ii) incomplete height history. After these, 78 participants remained. Since the participants were minors, we obtained parental consent before the study. This study was approved by the ethics committee of the Faculty of Sport Sciences, Waseda University (2016-098).

### 2.2. Procedure and Data Collection

We collected an elementary school growth chart between 6 and 11 years of age for each of the participants in 0.1 cm increments, which had been measured by a standard stadiometer. In addition, we measured the height with barefoot of each participant every six months, using a stadiometer between April/May of the first year (12 to 13 years old) and April/May of the third year (14 to 15 years old) of junior high school—a total of five measurements. Three examiners (TT, WS, TM) performed height measurements on eight adult participants twice on each day over a two-day period, indicating intra- and inter-rater reliability of 0.99 for both.

We use an AUXAL 3.1 program (AUXAL 3.1, Scientific Program International, Skokie, IL, USA), and a triphasic generalized logistic (BTT) model was used [17,18] to evaluate the indicators of each participant`s growth tempo and maturity status from the calculated growth curve. Subsequently, we extracted three events from the growth curve that was generated by the AUXAL 3.1 program at which the growth tempo changed: Take Off Age (TOA) when the annual height increase begins to increase, Peak Height Velocity Age (PHVA), and Final Height Age (FHA). In addition, we confirmed in our analysis of seven adult males (aged 20 to 22 years) whether the height data obtained in this study from 6 to 14 years of age accurately predicted their subsequent height at FHA.

As a result, no difference was found between the two groups (paired *t*-test; *p* = 0.635), with a mean of 171.49 ± 3.98 cm for the measured height and 171.59 ± 3.52 cm for the predicted height.

The length per one-year-interval (horizontal axis) and per 1 cm of height (vertical axis) from the origin in plane coordinates indicated by the AUXAL 3.1 program were calculated using Image J (National Institute of Health Bethesda, MD). Then, the height at TOA and PHVA was estimated by the ratio of their length from the origin based on the TOA and PHVA automatically indicated by the AUXAL 3.1 program. FHA was predicted as when the annual height increase was less than 1 cm, and height at FHA was predicted in the same way as described above. In addition, the period and height increase from TOA to PHVA and TOA to FHA was calculated by subtracting each event. Next, in order to calculate the growth tempo, the height increase from TOA to PHVA and FHA was divided by the period and expressed as Growth Tempo 1 and 2, respectively. The calculation formula is as follows:

(1) Growth tempo 1 = height increase/period from TOA to PHVA;

(2) Growth tempo 2 = height increase/period from TOA to FHA.

### 2.3. Maturity Status Classification

Z-scores were used to classify the maturity status on PHVA: middle maturation, z-score between −1.0 and +1.0; late maturation, z-score greater than +1.0; early maturation, z-score below −1.0 [10].

### 2.4. Statistics

Descriptive data are reported as averages, standard deviations, and 95% confidence intervals for TOA, PHVA, and FHA; height at TOA, PHVA, and FHA; the period from TOA to PHVA and TOA to FHA; height increase from TOA to FHA in early maturation, middle maturation, and late maturation groups, respectively. Then, to verify the normality of the data, the Shapiro–Wilk test was applied. Maturity-status-based comparisons of the timing and height at TOA, PHVA, and FHA; the period and height increase from TOA to PHVA and TOA to FHA; and Growth Tempo 1 and 2 were performed with one-way analysis of variance (ANOVA) among the three groups. When ANOVA showed a significant group effect, post hoc Bonferroni tests were performed. In addition, analysis of covariance (ANCOVA) was conducted regarding growth tempo to minimize the influence of body size differences between maturity levels shown by Iuliano-Burns [19] by entering height at TOA and PHVA into Growth Rate 1 and height at TOA and FHA into Growth Rate 2. For all tests, statistical analyses were performed using SPSS for Windows (IBM SPSS version 24.0; SPSS Inc., Chicago, IL, USA), and a *p*-value < 0.05 was used to determine statistical significance.

## 3. Results

Table 1 shows the descriptive data of maturity status in the early, middle, and late maturation groups. The overall PHVA was 13.29 ± 0.11 years, and the number of participants in the three groups was 13 in the early maturation, 55 in the middle maturation, and 10 in the late maturation groups. The TOA, PHVA, and FHA, and the period from TOA to PHVA were significantly different between the groups (early < middle < late; *p* < 0.05). Additionally, the early maturation group had lower TOA and FHA than the middle and late maturation groups. The height increase from TOA to FHA was larger in the early maturation than in the late maturation group.

**Figure 1:**
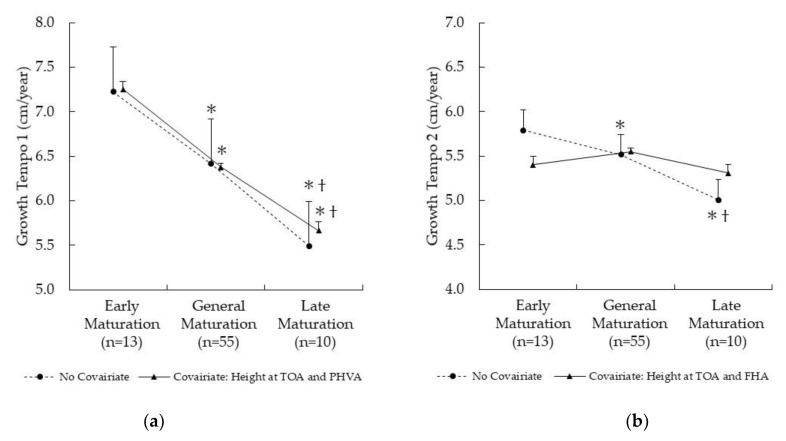
Difference of Growth Tempo among Early, Middle and Late Maturation: (**a**) Growth Tempo 1 (from Take Off Age to Peak Height Velocity Age); (**b**) Growth Tempo 2 (from Take Off Age to Final Height Age); *: vs. Early maturation; †: vs. Middle maturation.

Figure 1 shows the differences in Growth Tempo 1 (Figure 1a) and 2 (Figure 1b) among the early, middle, and late maturation groups. Although there was a significant difference between the three groups under the condition of no covariance for both Growth Tempo 1 and 2, there was no difference between the groups in Growth Tempo 2 when accounting for the covariants (height at TOA and FHA), and no change was observed when entering the covariants (height at TOA and PHVA) in Growth Tempo 1.

## 4. Discussion

This study investigated differences in the growth tempo between early, middle, and late maturation. The main finding was that the early maturation group had a higher growth tempo from TOA to PHVA than the middle and late maturation groups. The PHVA acquired as the standard for maturity status was 13.29 ± 0.11 years, which is close to what has been reported previously for Japanese subjects [2,3,20]. However, Nariyama et al. [21] reported that the PHVA of Japanese soccer players was 13.65 ± 1.09 years, which is later than in other sports (Baseball: 13.10 ± 0.96; Basketball ± 12.84 ± 1.12; Volleyball: 13.17 ± 0.80 years). It is possible that the difference in the PHVA may be related to the participation. Since the early maturation group had lower height at TOA, PHVA, and FHA than the middle and late maturation groups in this study, we entered the height at each event as a covariant in the ANCOVA. As a result, this did not alter the results for Growth Tempo 1 between early, middle, and late maturation, but for Growth Tempo 2, the group differences observed in the ANOVA were eliminated. Therefore, we can infer that the rapid growth tempo was caused by a height increase over a short period, considering that the early maturation group had a shorter interval from TOA to PHVA than the middle and late maturation groups, but there was no significant difference in increased height. Previous research reported a growth tempo from TOA to PHVA of 4.83 ± 1.61 cm per year and a PHVA of 14.26 ± 1.00 years [22]. Together with the overall score of Growth Tempo 1 from our study (6.43 ± 0.67 cm per year and PHVA of 13.29 ± 0.11 years), it suggests that growth tempo is rapid in early maturing children.

Growth and maturation are potential risk factors for injury [23], which may be explained by the fact that the annual height increase changes depending on the timing of maturity during the growth spurt period. Previous studies that investigated the relationship between changes in height increase during the growth spurt period and the occurrence of injuries reported that an increase in leg length and height were significantly associated with the risk of injury in athletes [15], soccer players [24], and ski racers [16]. However, a systematic review concluded that there was insufficient evidence to support a causal relationship between growth, maturation, and injury in adolescence [23]. Subsequent research with young soccer players has shown that players aged 10–12 years who sustained overuse injuries had also had a greater increase in height and leg length [24], and that growth-related injuries occurred more often at an earlier stage of growth [25]. Furthermore, given that overuse injuries such as tendinopathy and groin pain are more common in young soccer players who mature early [26], the results of our study suggest that rapid early maturation growth until PHVA may be associated with a higher occurrence of injuries. In addition, adolescents around the PHV period have been known to experience a decrease in skills and performance due to “adolescent awkwardness,” which is a result of the reduced coordination that may follow the rapid changes in limb length [1,12,27]. Although many studies have focused on the timing of maturity since late-maturing athletes are at a relative disadvantage in body size compared to early maturing athletes even at the same age, it is possible that athletes with a higher growth tempo could be at a disadvantage since they may take longer to adapt relative to their own growth. However, no research has investigated how growth tempo indicated as the rapidity or gradualness of own growth could influence skill and performance in sport. Further research may be needed that focuses on the developmental disadvantages of athletic performance to which precocious athletes could be exposed.

The PHVA of Japanese subjects is known to be earlier than that of Europeans [28,29] and North Americans [30]. Therefore, the growth tempo presented by adolescents in other countries may be more moderate than the results presented here. It should also be noted that there may be differences in the growth processes related to race.

Finally, an important issue that could be considered both a strength and a limitation of this study is measurement frequency during adolescence. It is conceivable that more frequent growth measurements during the adolescent growth spurt could have provided a more accurate estimate of actual age in PHV. Since those who were able to collect complete height history and measurement data for all 11 times from age 6 to 14 were recruited for the analysis, the accuracy of the growth spurt was considered to be guaranteed. Although it is not possible to specify the extent to which the height at FHA and FHA differed from the predicted values because we could not track the height after 15 years old, it did not deviate greatly from the height at FHA of the Japanese [31]. In addition, technical errors in height data collected using height records could not be estimated. Since this study only examined boys, future research on girls is also needed. In addition, as growth tempo is a new perspective, it can become a more useful indicator when findings from various countries, races, and sports are accumulated in the future. It must be noted, however, given the racial differences in growth and maturation, concerning the application of the results of this study, that they are only for Japanese.

## 5. Conclusions

The adolescent boys in the early maturation group grew rapidly from TOA to PHVA in a shorter period compared to those in the middle and late maturation groups. To improve athletic performance, prevent injuries, and find talent, the time of maturity and growth tempo should be evaluated. While evaluating as many high potential young athletes as possible, it is necessary for the coaches, trainers, and scientists to not only evaluate late maturation without overlooking them as has been discussed in the past but also pay attention to the rapidity of growth in early maturing individuals.

## Figures and Tables

**Table 1:** Descriptive data of maturity status in early, middle, and late maturation groups.

	Average	±	Standard deviation	95% CI ^1^			
Early maturation (*n* = 13)							
TOA ^2^ (years old)	8.87	±	0.14	8.57	-	9.18	
Height at TOA (cm)	132.32	±	1.78	128.44	-	136.21	
PHVA ^3^ (years old)	11.60	±	0.12	11.33	-	11.87	
Height at PHVA (cm)	152.16	±	1.48	148.94	-	155.39	
FHA ^4^ (years old)	15.32	±	0.09	15.12	-	15.51	
Height at FHA (cm)	169.75	±	1.44	166.62	-	172.88	
Period from TOA to PHVA (years)	2.73	±	0.10	2.51	-	2.94	
Period from TOA to FHA (years)	6.45	±	0.17	6.08	-	6.81	
Height Increase from TOA to PHVA (cm)	19.84	±	1.06	17.52	-	22.16	
Height Increase from TOA to FHA (cm)	37.42	±	1.40	34.37	-	40.48	
Middle maturation (*n* = 55)							
TOA (years old)	10.34	±	0.07	10.20	-	10.47	*
Height at TOA (cm)	138.25	±	0.79	136.67	-	139.84	*
PHVA (years old)	13.44	±	0.07	13.29	-	13.58	*
Height at PHVA (cm)	158.13	±	0.67	156.79	-	159.46	*
FHA (years old)	16.72	±	0.06	16.59	-	16.85	*
Height at FHA (cm)	173.50	±	0.66	172.18	-	174.81	*
Period from TOA to PHVA (years)	3.10	±	0.03	3.04	-	3.16	*
Period from TOA to FHA (years)	6.38	±	0.03	6.32	-	6.45	
Height Increase from TOA to PHVA (cm)	19.87	±	0.28	19.31	-	20.44	
Height Increase from TOA to FHA (cm)	35.24	±	0.47	34.31	-	36.18	
Late maturation (*n* = 10)							
TOA (years old)	11.34	±	0.10	11.10	-	11.58	*†
Height at TOA (cm)	139.86	±	1.79	135.80	-	143.92	*
PHVA (years old)	14.72	±	0.11	14.48	-	14.97	*†
Height at PHVA (cm)	158.45	±	1.36	155.37	-	161.52	*
FHA (years old)	17.83	±	0.12	17.55	-	18.11	*†
Height at FHA (cm)	172.36	±	1.15	169.76	-	174.96	†
Period from TOA to PHVA (years)	3.38	±	0.06	3.26	-	3.51	*†
Period from TOA to FHA (years)	6.49	±	0.06	6.34	-	6.63	
Height Increase from TOA to PHVA (cm)	18.58	±	0.48	17.49	-	19.67	
Height Increase from TOA to FHA (cm)	32.50	±	0.76	30.78	-	34.22	†

^1^ 95% confidence interval; ^2^ Take Off Age; ^3^ Peak Height Velocity Age; ^4^ Final Height Age. *: vs. Early Maturation; †: vs. Middle Maturation (*p*-value < 0.05 for ANOVA post hoc Bonferroni tests).

## Data Availability

The data presented in this study are available on request from the corresponding author and team director.
